# Non-Vaccine Serotype Replacement and Subdominant Persistence of Vaccine Types in Nepalese Infants Following PCV10 Introduction

**DOI:** 10.3390/vaccines14010073

**Published:** 2026-01-08

**Authors:** Fleurette Mbuyakala Domai, Dhruba Shrestha, Raj Kumar Shrestha, Monika Thimi, Desmond Opoku Ntiamoah, Yumiko Hayashi, Chris Smith, Yoshinao Kubo, Shunmay Yeung, Motoi Suzuki, Konosuke Morimoto, Koya Ariyoshi, Bhim Gopal Dhoubhadel

**Affiliations:** 1 Department of Clinical Medicine, Institute of Tropical Medicine, Nagasaki University, Nagasaki 852-8523, Japan; 2 Program for Nurturing Global Leaders in Tropical and Emerging Communicable Diseases, Graduate School of Biomedical Sciences, Nagasaki University, Nagasaki 852-8523, Japan; 3 National Dracunculosis Eradication Program, Kinshasa 01204, Democratic Republic of the Congo; 4 Siddhi Memorial Hospital (For Women and Children), Bhaktapur 44800, Nepal; 5 School of Tropical Medicine and Global Health, Nagasaki University, Nagasaki 852-8523, Japan; 6 Department of Clinical Research, London School of Hygiene & Tropical Medicine, London WC1E 7HT, UK; 7 Department of Infectious Disease Epidemiology, London School of Hygiene & Tropical Medicine, London WC1E 7HT, UK; 8 Department of Respiratory Infections, Institute of Tropical Medicine, Nagasaki University, Nagasaki 852-8523, Japan

**Keywords:** *Streptococcus pneumoniae*, pneumonia, pneumococcal conjugate vaccine (PCV), multi-serotype co-colonization, high-resolution molecular surveillance, dominance hierarchy, ecological shift, Nepal

## Abstract

**Background:** *Streptococcus pneumoniae* is a leading cause of child mortality in Nepal despite the introduction of the 10-valent pneumococcal conjugate vaccine (PCV10). Vaccine effectiveness is threatened by the emergence of non-vaccine serotypes (NVTs) and the multiple serotypes carriage which often fail to be detected by traditional methods. We aimed to study changes in serotype distribution before and after PCV10 immunization among infants, including serotype dominance in Nepalese infants in the post-vaccine era. **Methods:** We enrolled infants in a longitudinal cohort study (2020–2022) conducted in Bhaktapur, Nepal. Nasopharyngeal swabs were collected before PCV10 dose 1 (6 weeks) and at 9 and 12 months post-immunization. We used a sensitive nanofluidic qPCR platform to detect multiple serotypes and establish their hierarchy by quantifying the bacterial load of each strain. Inverse Probability Weighting (IPW) adjusted risk factor analysis was used to account for loss to follow-up. **Results:** PCV10 successfully reduced vaccine-type (VT) carriage, declining sharply from 32.8% at 6 weeks to 4.8% at 12 months. VTs were pushed from being the dominant strain to occupying subdominant roles in co-colonization. Conversely, NVTs rapidly filled the vacated niche, showing a significant increase in their dominant status (*p* < 0.001). The most common replacing NVTs that rose to dominance were 35B, 19A, 6C/6D, and 15B/15C. Significant risk factors for carriage included older infancy (aOR 3.4, 95%CI: 2.6–4.5 at 9 months), a household kitchen in the living area (aOR 1.4, 95%CI: 1.0–1.9), and winter (aOR 1.7, 95%CI: 1.5–2.7) and pre-monsoon seasons (aOR 2.0, 95%CI: 1.5–2.8). **Conclusions:** While PCV10 reduced overall VT circulation, the persistence of VTs in subdominant niches creates a continuous reservoir for potential re-emergence and antibiotic resistance. This clear hierarchical shift in dominance towards NVTs underscores the urgent need for a public health strategy that includes the adoption of a higher-valent PCV to provide broader protection, and interventions targeting environmental risk factors are essential to sustain long-term reductions in pneumococcal colonization.

## 1. Introduction

*Streptococcus pneumoniae* (pneumococcus) remains a significant global health threat. This bacterium is responsible for nearly one million child deaths annually, with the highest burden concentrated in Asia and Sub-Saharan Africa [1,2,3]. Nasopharyngeal carriage of the bacteria is a crucial step for both transmission and diseases [4]. Studies show that children in lower-income countries are colonized earlier in life, carry the bacteria more frequently, and are more likely to harbor multiple serotypes simultaneously [4,5]. While Pneumococcal Conjugate Vaccines (PCVs) have been highly effective in reducing disease by limiting the carriage of vaccine-targeted serotypes, their success is consequently challenged by “serotype replacement”, a phenomenon where non-vaccine serotypes emerge to fill the vacated ecological niche [6,7]. This is therefore a key concern in Nepal, which introduced the 10-valent PCV (PCV10) into its national immunization program in 2015 [8]. Thus, effective surveillance is essential to monitor these shifts and assess the impact of vaccination programs, particularly in low- and middle-income countries (LMICs), even after PCV introduction [9].

However, traditional culture-based methods have a significant limitation: they often fail to detect subdominant serotypes within individuals carrying multiple serotypes simultaneously [10,11]. The presence of these subdominant populations is not an incidental finding; it represents a persistent and significant public health reservoir. These low-density strains pose a continuous threat because they fuel transmission within the community and are positioned to harbor genes for antibiotic resistance. Furthermore, this pool of persistent serotypes retains the potential to rapidly gain a competitive advantage, leading to the re-emergence of pneumococcal diseases caused by serotypes included in the vaccine, should vaccine-induced pressure diminish, or ecological conditions change. Ultimately, their maintenance severely impacts the long-term effectiveness of current vaccination programs. This is particularly critical in the post-vaccine era, as subdominant serotypes may be under-diagnosed by traditional methods [11].

Due to the scarcity of data on multi-serotype carriage, major concerns remain about their effect on pneumococcal population dynamics. Therefore, elucidating the prevalence and distribution of these subdominant populations is essential for informing future vaccine strategies. Despite this, there is a significant lack of longitudinal cohort data tracking subdominant serotypes following PCV10 introduction.

To address this knowledge gap, our study is the first to use a high-resolution molecular approach to longitudinally track the distribution, dominance, and bacterial load of both vaccine and non-vaccine serotypes in Nepalese infants. By providing a detailed view of serotype competition over the first year of life, we offer crucial insights into the real-world impact of vaccination on the pneumococcal ecosystem, thereby informing future strategies to sustain disease reduction.

## 2. Methods

### 2.1. Study Design and Setting

We conducted this longitudinal cohort study in Bhaktapur, Nepal, focusing on Siddhi Memorial Hospital (SMH). SMH is the only dedicated pediatric hospital in the district [12]. The hospital is a 50-bed facility that functions as a critical care center for children in the region. SMH offers a comprehensive range of pediatric and maternal healthcare services, including an outpatient department (OPD) with roughly 15,000 visits annually, a busy emergency department, a 50-bed inpatient department (IPD) recording around 1500 annual admissions, and a neonatal intensive care unit. Other essential services provided include routine immunization, gynecology/obstetrics, and dental care [12].

### 2.2. Study Period and Population

The study took place from February 2020 to December 2022. All healthy infants (defined as those without any acute illness at the time of enrolment) aged 6 weeks attending the immunization clinic for their first PCV10 dose were eligible for inclusion and approached for the recruitment. Infants with a history of antibiotic use in the preceding month, congenital abnormalities affecting the mouth, nose, or nasopharynx, and those for whom parents declined participation or withdrew consent were excluded. Participants were enrolled using systematic random sampling. A list of eligible infants was generated for each immunization day. To ensure randomness, a random number generator was used to select a starting point from this list, and every third infant thereafter was approached for enrolment.

### 2.3. Data Collection

**Figure A1:**
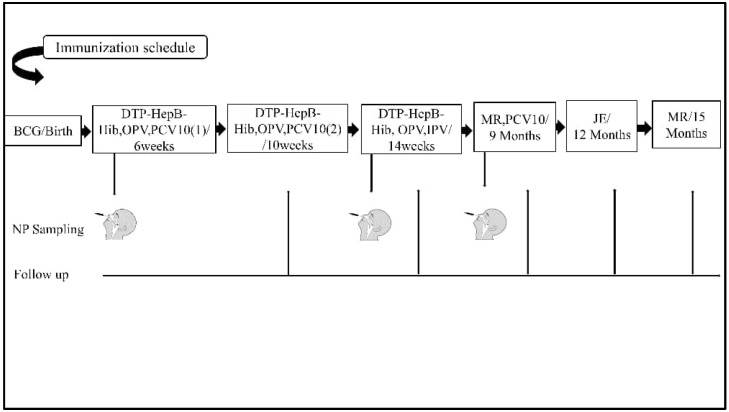
Immunization schedule and nasopharyngeal sampling timeline. Schematic of PCV10 doses and the timing of swab collection at 6 weeks, 9 months, and 12 months.

After obtaining informed consent from parents or guardians, data were collected using a standardized questionnaire on a tablet via the Open Data Kit (ODK) platform. Information was collected on demographics, living conditions (including crowding and indoor pollution), antenatal history, and any respiratory illnesses. Follow-up visits were conducted at 10 weeks, 14 weeks, 9 months, 12 months, and 15 months, aligning with routine vaccination schedules. During follow-up visits, illness histories and general examinations were conducted. Nasopharyngeal swabs were collected at 6 weeks (before the first PCV10 dose), 9 months (after two doses), and 12 months (after three doses) (Appendix A Figure A1).

### 2.4. Sample Size

The sample size was calculated to detect an epidemiologically significant, 10-percentage-point increase in non-vaccine pneumococcal serotype (NVT) carriage at 12 months, from an assumed baseline of 40% [13] to 50%. To compensate for a projected 40% loss to follow-up, a minimum of 318 participants needed to be enrolled. To further enhance the precision of our estimates and ensure adequate power for multivariable risk factor models, we established a final enrollment target of 600 infants.

### 2.5. Laboratory Analysis

#### 2.5.1. Sample Collection and Processing

Nasopharyngeal samples were collected by a skilled nurse according to World Health Organization recommendations and stored appropriately before analysis [14]. After collection, nasopharyngeal swabs were immediately placed in tubes containing 1 mL of Skim Milk, Tryptone, Glucose, and Glycerol (STGG) transport medium and stored at −80 °C within 8 h of collection [14]. DNA was extracted at SMH using the QIAamp DNA Mini Kit (Qiagen, Hilden, Germany) according to the manufacturer’s protocol and shipped on dry ice to Nagasaki University, where they were stored at −80 °C for downstream analyses.

#### 2.5.2. Pneumococcal Detection and Serotyping

Pneumococcal detection and serotyping followed previously established methods [11]. Briefly, DNA was extracted from STGG and analysed using lytA real-time quantitative PCR (qPCR) to identify pneumococci and quantify their density, expressed in genome equivalents per millilitre (GE/mL) [11].

For bacterial identification, we prepared a positive control stock solution with a volume of 100 μL and a concentration of 13.5 ng/μL, using 5 μL of DNA from all isolated serotypes included in the serotyping analysis, which contained 3 × 10^6^ copies. We then performed a 10-fold serial dilution of this stock solution to determine the lower limit of detection, set at 30 cycles [11].

Pneumococcal presence was first determined by lytA real-time qPCR. Samples with a lytA cycle threshold (Ct) value < 30 were classified as pneumococcus positive [11]. For serotype assignment in the nanofluidic qPCR assay, we applied a more stringent threshold and considered a serotype to be present only if its individual Ct value was <25, in line with previously described methods [11]. Samples with lytA Ct < 30 but without any serotype-specific signal < 25 were classified as pneumococcal positive but non-typeable. To define serotype dominance, we relied on the quantitative nature of the nanofluidic qPCR. The Ct value has a negative correlation with the starting quantity of the target DNA [11]. Thus, a lower Ct value signifies a higher bacterial load. In samples where multiple serotypes were detected, the serotype with the lowest Ct value was designated as the dominant serotype [11]. We then ranked the remaining serotypes based on their Ct values to identify the second and third most dominant serotypes, providing a quantifiable and objective measure of relative abundance in cases of co-colonization. Molecular serotyping was performed using the X9 nanofluidic real-time PCR system, to identify up to 70 different pneumococcal serotypes as previously described [11] (Supplemental Material p2). The study focused on the three serotypes with the lowest Ct values to identify the predominant serotypes.

### 2.6. Data Analysis

#### Descriptive Analyses

Categorical data were reported as frequencies and percentages, and continuous data as medians with interquartile ranges (IQR). For this analysis, pneumococcal serotypes were primarily categorized based on their inclusion in the PCV10 formulation. Serotypes were classified as either PCV10 vaccine types (VTs) or non-PCV10 serotypes. The non-PCV10 serotypes category includes all serotypes not in PCV10, which include those added in later vaccines (PCV13, PCV15, PCV20) as well as serotypes not in any vaccine formulation (true NVT) and non-typeable (NT) isolates. Serotypes included in different conjugate vaccines are shown in Table 1.

A methodological limitation was the inability of our molecular methods to differentiate between serotypes 6A and 6B due to their high genetic similarity [11]. This posed a classification challenge, as 6B is a PCV10 serotype, while 6A is a non-PCV10 serotype and a PCV13 target associated with replacement [15]. To ensure the transparent calculation of the proportions of PCV10 and non-PCV10 serotypes, all isolates identified as 6A/6B were treated as a separate “6A/6B group” in the primary analysis. The study analysed how the proportions of these categories (PCV10, non-PCV10, and the 6A/6B group) changed over time to understand trends in serotype carriage.

### 2.7. Statistical Analysis

To mitigate potential selection bias from participant attrition in this longitudinal cohort, we used Inverse Probability Weighting (IPW) [16]. First, we compared baseline characteristics between infants who completed the study and those lost to follow-up to identify predictors of attrition. Next, we developed a multivariable logistic regression model to calculate each participant’s probability of retention at 9 and 12 months. The inverse of these probabilities served as weights in all subsequent analyses. To assess risk factors for pneumococcal carriage, we applied these weights to logistic regression models, with generalized estimating equations (GEEs), to examine associations between pneumococcal carriage, multiple pneumococcal carriage and potential risk factors (Supplemental Material p3). Results were reported as odds ratios (ORs) with 95% confidence intervals (CIs). Pneumococcal density was log10-transformed and compared between samples with a PCV10 serotype as the first serotype and those with a non-PCV10 serotype, between single and multiple serotype carriage, as well as 6A/6B serotypes over time using Mann–Whitney U test. Data were processed in Microsoft Excel (Version 2408, Microsoft Corp., Redmond, WA, USA) and analysed in Stata 15·0. (StataCorp LLC, College Station, TX, USA). A *p*-value ≤ 0.05 was considered statistically significant.

## 3. Results

### 3.1. Characteristics of Study Participants at Enrolment

**Figure 1:**
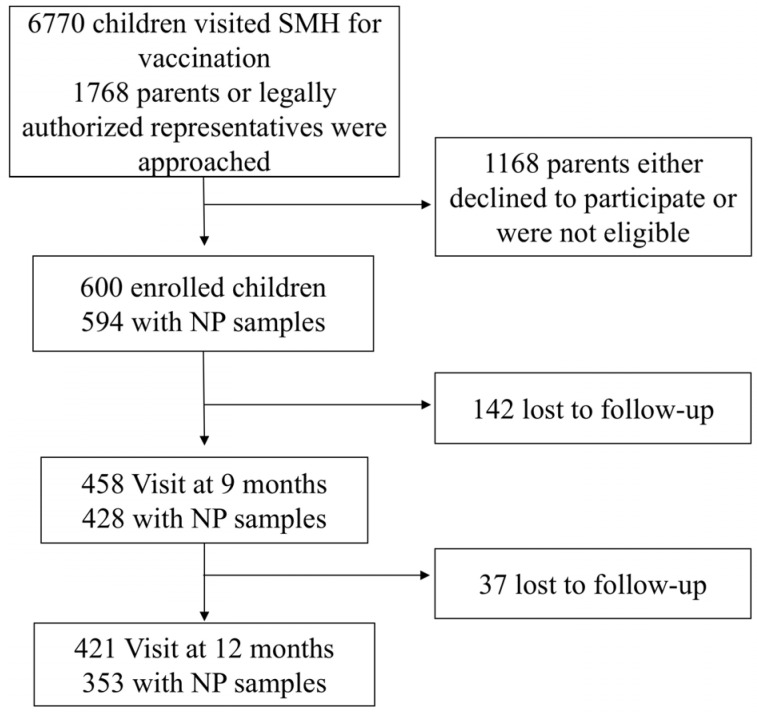
Study Flow Chart. This chart illustrates the enrolment and follow-up procedures for the pneumococcal carriage study conducted at Siddhi Memorial Hospital (SMH) from February 2020 to December 2022. NP, nasopharyngeal.

A total of 600 children were enrolled in the study with 594 (99%) providing both clinical data and nasopharyngeal swabs at enrolment. Follow-up rates declined over time, with 428 (71%) children providing samples at 9 months and 353 (59%) children at 12 months (Figure 1).

Table 2 summarizes the characteristics of the study participants at enrolment. The cohort had a slight male predominance 319/594 (54%) and a median age of 1.5 months (IQR: 1.4–1.6). Most infants were born at term 583/594 (99%) with a birth weight above 2500 g 535/594 (91%). The largest ethnic group was Newar 359/594 (60%), followed by Tamang 116/594 (20%). Most mothers were between 20 and 35 years old 522/594 (88%), with a median age of 29 years (IQR: 26–32). Over half of the mothers had secondary-level education 308/594 (52%), and 40% had higher education. Regarding overcrowding and indoor pollution characteristics, 56% of families had fewer than five members (329/594), while 42% lived in households with 5–10 members. 42% of households reported the presence of a smoker (250/594). A significant majority (78%) of households had a separate kitchen room, while 22% cooked in the living room (132/594). The majority of children were exclusively breastfed, 428/594 (72%).

To assess potential bias due to loss to follow-up, baseline characteristics at enrolment (6 weeks) were compared between participants who completed all three scheduled visits and those who were lost to follow-up at either the 9-month or 12-month time point. Appendix A Table A1 presents this comparison. No statistically significant differences were observed between the retained and lost-to-follow-up groups for the all the baseline characteristics, except that there was a borderline difference in retention status by household size (*p* = 0.05) and source of income (*p* = 0.06).

### 3.2. Temporal Changes in Pneumococcal Carriage Prevalence

Of the 1375 nasopharyngeal swabs analysed, 567 (41.2%) were positive for pneumococcus. Pneumococcal carriage prevalence increased from 30·1% (179/594, 95% CI: 26.4–33.8%) at enrolment to 54·9% (235/428, 95% CI: 50.2–59.6%) at 9 months, then decreased to 43·3% (153/353, 95% CI: 38.1–48.5%) by 12 months. Multiple serotype carriage followed a similar trend, with 40·7% (69/172, 95% CI: 33.3–48.1%) of carriers at enrolment, 47.4% (109/230, 95% CI: 40.9–53.9%) at 9 months, and 33.1% (47/142, 95% CI: 25.3–40.9%) at 12 months carrying more than one serotype (Table 3).

### 3.3. Serotype Distribution

**Figure 2:**
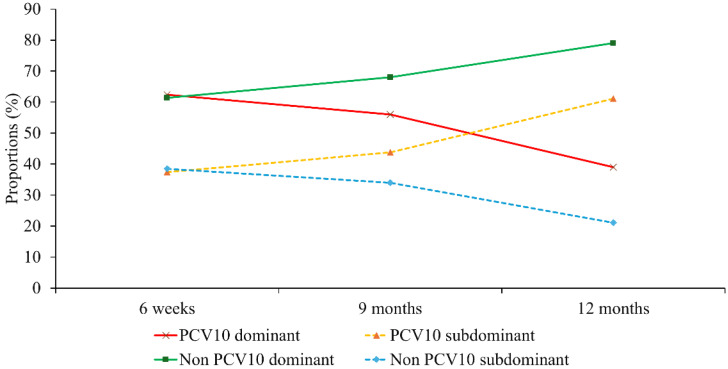
Trends in dominant and subdominant pneumococcal carriage. Longitudinal proportions of dominant and subdominant vaccine types (PCV10) and non-vaccine types (Non-PCV10) across the three study time points.

**Figure A2:**
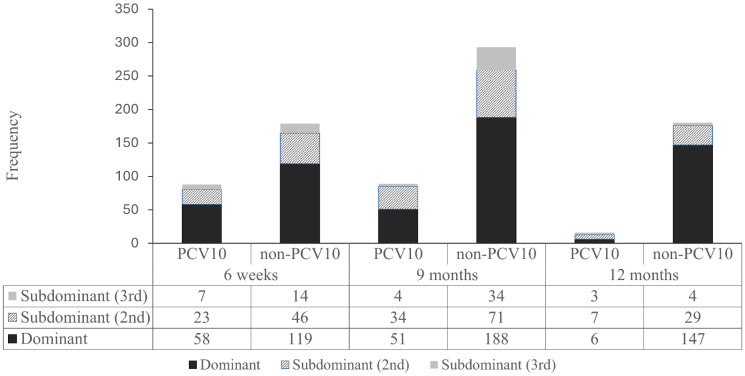
Distribution of PCV10 and non-PCV10 serotypes by dominance. Proportional representation of dominant and subdominant serotypes within the PCV10-VT and Non-PCV10 categories across all time points.

Nanofluidic qPCR revealed a high prevalence of multiple-serotype carriage, allowing classification of pneumococcal vaccine serotypes by dominance based on Ct values and relative abundance. As shown in Appendix A Figure A2, in addition to the serotype distribution of dominant serotypes, serotype distributions of a substantial number of second most dominant serotypes and non-negligible number of third most dominant serotypes were disclosed. Overall, non-PCV10 serotypes were predominant across all time points, including even before the immunization (6 weeks). PCV10 serotypes appeared less frequently, particularly in dominant and second dominant positions, suggesting reduced nasopharyngeal colonization of vaccine-covered serotypes post-vaccination. At 6 weeks, the proportion of dominant PCV10 serotype was nearly identical to that of non-PCV10 serotype (57/91, 62.6% vs. 105/174, 60.3%), and the same pattern was observed for the proportion of subdominant PCV10 serotypes (34/91, 37.3% vs. 69/174, 39.6%). However, these proportions diverged over time, depending on whether the serotype was included in PCV10. The proportion of PCV10 serotypes detected as subdominant increased over time from 37.3% (34/91) at 6 weeks, to 43.8% (39/89) at 9 months, and 61.1% (11/18) at 12 months (*p* = 0.17 by chi square test). In contrast, the proportion of non-PCV10 serotypes detected as subdominant showed a decreasing trend from 39.6% (69/174) at 6 weeks, 34,2% (92/269) at 9 months, and 22.4% (34/170) at 12 months (*p* < 0.001) (Figure 2).

The 6A/6B serotype group, when analysed, exhibited sustained high-density carriage throughout the study period. At 6 weeks, this group was dominant, detected in 83.3% (5/6) of its samples, with only 16.7% (1/6) found in a subdominant role. Although the total number of 6A/6B detections peaked at 9 months, it remained highly dominant in 69.2% (9/13) of its samples. By the 12-month follow-up, the group was entirely detected in the dominant position (100%, 6/6 samples) (*p* = 0.53 by chi square test).

**Figure 3:**
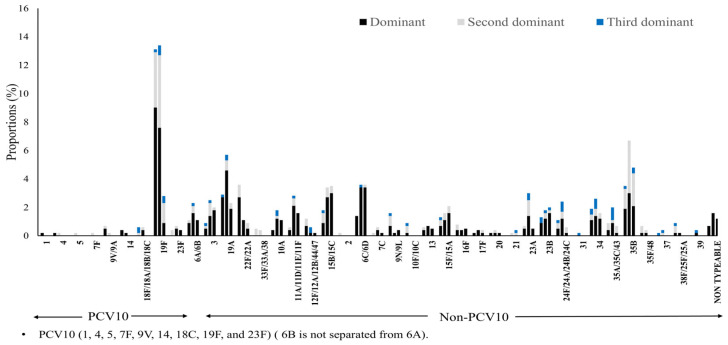
Serotype-specific distribution by dominance and time point. Proportions of individual dominant (black), second-dominant (gray), and third-dominant (blue). Each set of three stacked bars represents one specific point in time: the first set at 6 weeks, the second at 9 months, and the third at 12 months.

Figure 3 and Table A2 further illustrate the individual serotype-level distribution. The most frequently detected dominant serotype was 19F, a PCV10 serotype, followed by several non-PCV10 serotypes including 35B, 15B/15C, 6C/6D, and 19A. Notably, non-typeable pneumococci also appeared with increasing frequency. Intriguingly, the frequency of detection for certain serotypes varied considerably depending on whether subdominant serotypes were included in the analysis or not. For example, serotype 35B became one of the most detected serotypes when subdominant classifications were considered. Serotype 19F was still detected at 12 months but it was more often identified as subdominant serotype. On the other hand, the detection frequency of serotype 6C/6D remained stable across time points, and when detected, it was mostly classified as a dominant serotype. The 6A/6B group also featured prominently, being detected as dominant in 5 samples at 6 weeks, 9 samples at 9 months, and 6 samples at 12 months and as subdominant in 1 sample at 6 weeks and 4 samples at 9 months.

### 3.4. Proportions of Serotypes Covered by Different Vaccines

**Figure A3:**
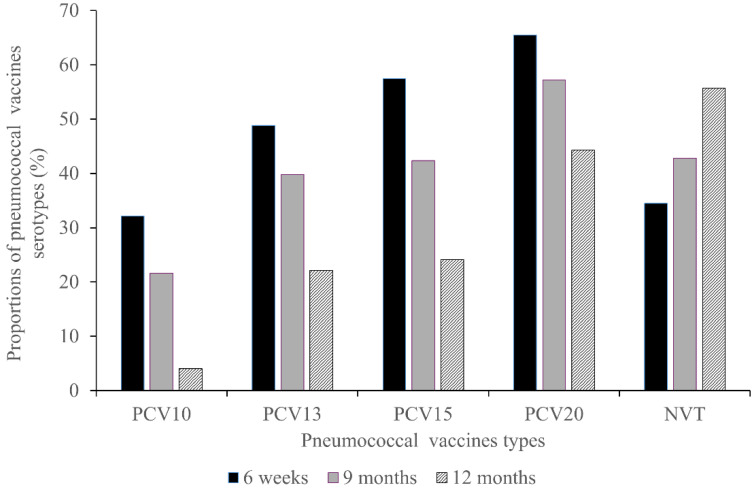
Coverage of pneumococcal conjugate vaccines. Proportions of circulating serotypes covered by PCV10, PCV13, PCV15, and PCV20 at 6 weeks, 9 months, and 12 months.

At 6 weeks of age (pre-vaccination), PCV10 serotypes accounted for approximately 32.8% of all pneumococcal serotypes detected. Coverage increased progressively with higher-valent vaccines: PCV13 covered 48.5%, PCV15 57.4%, and PCV20 65.5% of circulating serotypes. Non-vaccine types (NVTs) represented about 34% of all isolates. At 9 months (after two PCV10 doses), a notable decline was observed in PCV10-type carriage to 21.8%, while PCV13, PCV15, and PCV20 covered 38.9%, 42.4%, and 57.2% of detected serotypes, respectively. The proportion of NVTs increased to approximately 43%, suggesting early evidence of serotype replacement. At 12 months (post–vaccine schedule completion), PCV10-type carriage further decreased to 4.8%, indicating good impact on primary vaccine-type serotypes. However, higher-valent vaccines such as PCV13, PCV15, and PCV20 would have covered 22.5%, 24.2%, and 44.4% of detected serotypes, respectively. This substantial increase in potential coverage is primarily due to the targeting of emerging dominant serotypes, such as 19A and 3, which have replaced the PCV10-targeted strains and are established contributors to the regional clinical disease burden. The share of NVTs continued to rise, reaching about 55% of all isolates at this stage (Appendix A Figure A3).

### 3.5. Bacterial Load

**Figure 4:**
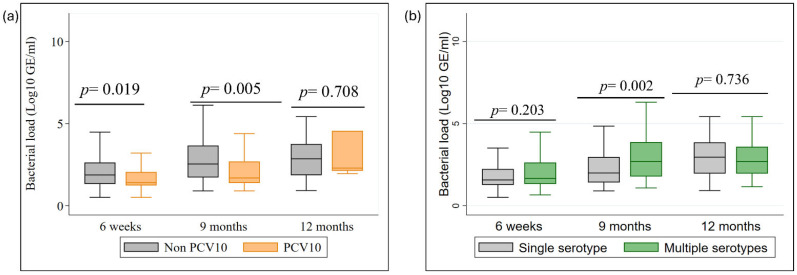
Bacterial density dynamics by serotype group and carriage type. (**a**) Comparison of genome equivalents (GE/mL) between PCV10 and Non-PCV10 serotypes; (**b**) Comparison of GE/mL between single and multiple serotype carriage across 6-week, 9-month, and 12-month time points.

**Figure A4:**
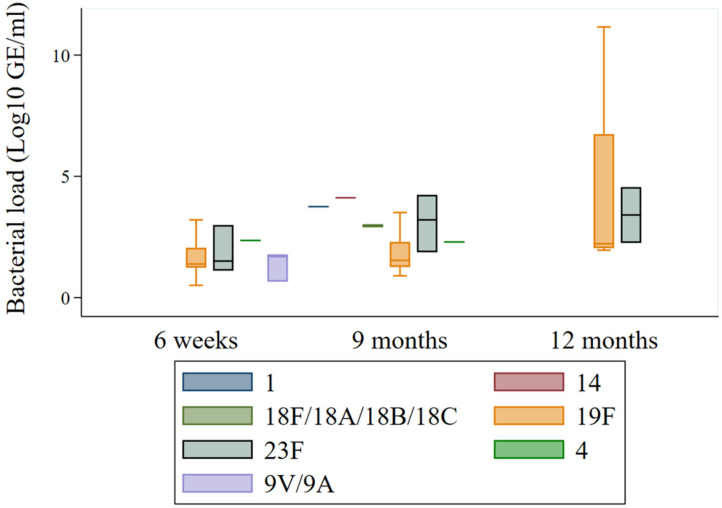
Serotype-specific bacterial load of PCV10 vaccine types. Boxplots representing the density (GE/mL) of individual PCV10 serotypes at 6 weeks, 9 months, and 12 months.

We compared bacterial load over time, as shown in Figure 4. Non-PCV10 serotypes consistently exhibited higher bacterial loads than PCV10 serotypes at all time points (Figure 4a). The difference was statistically significant at 6 weeks (*p* = 0.019) and 9 months (*p* = 0.005) but not at 12 months, likely due to the small number of cases of PCV10 serotype at 12 months and the presence of outlier cases, particularly cases of serotype 19F (n = 5) and 23F (n = 2) showed a relatively high bacterial load (*p* = 0.708) (Appendix A Figure A4). In Figure 4b, at 9 months, individuals with multiple serotypes had significantly higher bacterial loads compared to those with a single serotype (median: 3 log10 GE/mL, *p* = 0·002). This difference was not significant at 6 weeks and 12 months.

**Figure A5:**
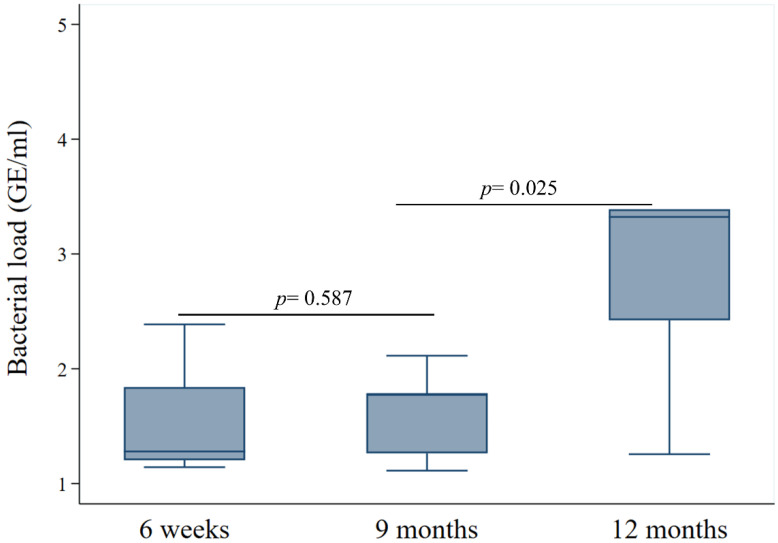
Bacterial load dynamics of the Ambiguous 6A/6B group. Density (GE/mL) of the 6A/6B serotype group at 6 weeks, 9 months, and 12 months.

The serotype Group 6A/6B also maintained high bacterial loads. Visually, the median density remained consistently high across all three time points, showing a trend of persistence rather than decline. There was no significant difference in bacterial load when comparing the 6A/6B group at 6 weeks versus 9 months (*p* = 0.59) but the difference was statistically significant when comparing 9 months versus 12 months (*p* = 0.02) (Appendix A Figure A5).

### 3.6. Factors Influencing Carriage Prevalence

Table 4 presents the logistic regression analysis of factors associated with pneumococcal carriage (N = 1375). The analysis used generalized estimating equations to account for repeated sampling from the same individuals, with all odds ratios adjusted by IPW to account for loss to follow-up. Pneumococcal carriage prevalence varied significantly with age, peaking at 9 months. Compared to infants at 6 weeks, the adjusted odds of carriage were significantly higher at 9 months (aOR: 3.4; 95% CI: 2.6–4.6) and at 12 months (aOR: 2.0; 95% CI: 1.5–2.7).

Environmental and seasonal factors also showed a significant association with carriage. Infants from households with a kitchen located in the living room had significantly higher adjusted odds of carriage (aOR: 1.4; 95% CI: 1.0–1.9) compared to those with a separate kitchen. Seasonality was a strong predictor of carriage. Compared to the monsoon season, the adjusted odds of carriage were significantly higher during the pre-monsoon (aOR: 2.0; 95% CI: 1.5–2.8) and winter seasons (aOR: 1.7; 95% CI: 1.5–2.7). No significant association was found between gender and pneumococcal carriage.

### 3.7. Multiple Serotype Carriage

Table 5 presents the logistic regression analysis of factors associated with multiple pneumococcal carriage in pneumococcal-positive samples (n = 567). The analysis used generalized estimating equations to account for repeated sampling, with all odds ratios adjusted by IPW to account for loss to follow-up. Among pneumococcal-positive samples, the odds of multiple carriage varied significantly with age. Compared to infants at 6 weeks, the adjusted odds of multiple carriage were significantly higher at 9 months (aOR: 1.7; 95% CI: 1.1–2.7). This association was not significant at 12 months (aOR: 1.0; 95% CI: 0.6–1.7). There was no statistically significant association between multiple carriage and gender, kitchen location, or seasons after adjusting for other factors. The aORs for these variables all crossed the null value of 1.0, indicating no significant effect on the odds of carrying multiple serotypes.

## 4. Discussion

We used a highly sensitive nanofluidic qPCR platform to conduct what is, to our knowledge, the first prospective birth cohort of Nepalese infants to longitudinally characterise dominant and subdominant pneumococcal serotypes and their densities at key immunisation milestones in the post-PCV10 era. Our central finding is that PCV10 vaccination pressure is associated with a clear ecological shift. The strongest evidence for this was a significant decline in the proportion of non-PCV10 serotypes found in subdominant roles (*p* < 0.001), as they rose to fill the dominant niche. Concurrently, we observed that VTs were pushed from dominant to subdominant roles. At 6 weeks (pre-vaccination), most detected VTs were dominant (approximately 62%). Following vaccination, the proportion of VTs detected as dominant fell to approximately 40% by 12 months. While the corresponding increase in the proportion of VTs found as subdominant was not statistically significant (*p* = 0.17), this proportional shift in dominance clearly illustrates the changing competitive landscape. This decline aligns perfectly with the vaccine’s expected effect in reducing carriage and transmission of dominant VTs strains [17].

Our data at the 6-week time point provide a baseline for our study cohort before they receive their first dose of PCV10. It is crucial to clarify, however, that this does not represent a true pre-vaccine era baseline for the broader population of Nepal. Our study was conducted from 2020–2022, approximately five years after Nepal’s national PCV10 program was initiated [8]. Consequently, the serotype distribution observed at 6 weeks already reflects a nasopharyngeal environment that has been shaped by five years of widespread vaccination. The low prevalence of VTs and the early dominance of non-PCV10 in our cohort even at this young age underscore the profound and rapid impact of the national immunization program on pneumococcal ecology [18].

Serotype 19F was the most detected subdominant VT in our cohort. This continued circulation, also seen in other settings [17,19,20], suggests that while the vaccine reduces 19F from achieving high dominant densities, it not eliminates it completely [21]. Several factors likely contribute to this observation [22]. For instance, 19F may require substantially higher antibody concentrations for protection compared to other VTs like 6B and 23F [22]. Voysey and colleagues estimated that the IgG concentration [22], (a proxy for carriage acquisition) was approximately 2.54 µg/mL for 19F, compared to only 0.50 µg/mL for 6B. Finally, inherent biological properties of serotype 19F, might also contribute to its resilience under vaccine pressure [23,24]. The continued circulation of 19F highlights the complexities of vaccine impact on mucosal colonization and underscores the importance of ongoing surveillance. This suggests “niche adaptation” rather than elimination. These subdominant vaccine-type serotypes thus represent a continuous reservoir, contributing to their ongoing circulation despite widespread vaccination and posing a challenge to the complete eradication of vaccine-targeted strains [25].

In contrast, non-PCV10 showed the opposite trend. Their subdominant forms significantly decreased as their dominant forms increased. This inverse relationship clearly underscores the competitive advantage non-PCV10 have gained by filling the niche VTs have vacated. The dominance of emerging serotypes like 35B, 15B/15C, 6C/6D,19A and 3 is particularly concerning, as they are increasingly associated with antibiotic resistance and mortality in the post-PCV era in different other regions [17,18,26,27].

Our longitudinal data complements and extends broader observations from other post-PCV10 settings. For example, Kandasamy et al. also documented a significant decline in VT carriage in Nepal, from 36.5% pre-PCV10 period to 10.3% post-PCV10, confirming the strong population-level impact of the PCV10 program [18]. This nuance also applies to pneumococcal density. Kandasamy et al. reported a modest decrease in median density, from 3.3 log10 GE/mL to 3.25 log10 GE/mL [18] which our data helps explain. We found that total density figures can be misleading, as they mask divergent, serotype-specific trends. Specifically, non-PCV10 consistently maintained higher bacterial loads than VTs. This is clinically critical, as a high bacterial load is a known risk factor for both transmission and invasive disease [28]. However, our high-resolution approach reveals a more intricate mechanism behind this decline. We demonstrate that this shift is not just about presence or absence, but about a change in competitive dynamics, whereby VTs are pushed into subdominant niches. Despite laboratory methodological differences between our studies the concordance on major trends strengthens the evidence, while our individual tracking provides novel insights.

Beyond these serotype dynamics, our study identified significant environmental and host predictors of carriage. Age was a key factor, with carriage peaking at 9 months (aOR: 3.4; 95% CI: 2.6–4.6). Increased in household social and environmental exposure during this developmental stage, could have led to more intense close contact and potentially poorer ventilation within households. While this increased contact likely plays a role, the subsequent reduction in carriage at 12 months (aOR: 2.0; 95% CI: 1.5–2.7) is likely multifactorial and may also be influenced by the completion of the PCV10 vaccination schedule and the natural maturation of the immune system.

Furthermore, strong environmental links were clear. Infants in households with a kitchen in the living room had 40% higher odds of carriage, suggesting a direct link between indoor air quality and colonization [29]. This conclusion is powerfully supported by the MSCAPE study in Malawian infants, which found a significant dose–response relationship between directly measured personal particulate matter with a diameter of ≤2.5 μm (PM_2.5_) exposure and the prevalence of nasopharyngeal *S. pneumoniae* carriage [29]. They observed a distinct difference in pollution exposure based on carriage status, children who were positive for pneumococcal carriage exhibited a significantly higher geometric mean PM_2·5_ exposure of 60·3 μg/m^3^ (95% CI 55.8–65.3) [29]. This exposure to pollutants from cooking fuels can impair mucosal defenses, making colonization easier [29,30]. This finding identifies a potential modifiable target for reducing pneumococcal disease risk.

Additionally, carriage was significantly higher during the pre-monsoon and winter seasons, consistent with global patterns for respiratory infections [31,32]. Seasonality is often attributed to complex interactions between environmental factors like temperature, humidity influencing pathogen survival and transmission, host behavior including increased indoor crowding during adverse weather, host physiology, and the co-circulation of other respiratory pathogens, particularly viruses, which can modulate susceptibility to bacterial colonization [31,33]. In contrast to overall carriage, factors influencing multiple carriage were less pronounced; here, only age was a significant predictor.

All findings from this study must be interpreted through the lens of the COVID-19 pandemic. The study period (2020–2022) was characterized by non-pharmaceutical interventions (NPIs), altered health-seeking behaviors, and profound disruptions to the circulation of typical respiratory viruses like influenza and RSV [34]. These viruses are known drivers of pneumococcal carriage and transmission. Therefore, the carriage prevalence (peaking at 54.9%) and the seasonal patterns observed (pre-monsoon and winter) may not be representative of a typical post-PCV10 era. The pandemic’s impact on social mixing and viral co-infection likely altered the pneumococcal competitive dynamics we observed, though the direction and magnitude of this effect are difficult to ascertain.

Taken together, our findings have clear implications for public health strategy in Nepal. The documented serotype replacement toward potentially more virulent non-PCV10 serotypes highlights the limitations of PCV10. The limited effectiveness against 6A [35,36] and the rise of serotypes like 19A and 3, which are included in PCV13 and are a known cause of pneumonia in Nepal [8], create an urgent need for action [15,37]. Our data strongly suggest a transition to a broader-coverage vaccine is necessary. While we acknowledge that carriage prevalence does not perfectly predict disease incidence, the high bacterial load associated with replacing NVTs in our cohort (Figure 4) serves as a critical indicator of increased transmission and invasive potential. Our results thus provide an immunological and ecological rationale for the adoption of higher-valency vaccines, as the serotypes rising to dominance in the nasopharynx mirror those responsible for clinical disease. While PCV13, PCV15 or another PCV10 such as PNEUMOSIL^®^ [38] would offer incremental benefits, our analysis indicates PCV20 would provide the most substantial advantage. This must be paired with continued, sensitive molecular surveillance to track these rapid ecological shifts and with public health interventions that address environmental drivers, such as indoor air quality.

This study has multiple strengths. Principally, it leverages a high-resolution molecular technique for quantifying multiple serotypes with high sensitivity. The longitudinal design allowed for temporal assessments across key immunization milestones, while the inclusion of density data provided mechanistic insights. Furthermore, conducting this study during the COVID-19 pandemic highlights the resilience of Nepal’s immunization program and provides a crucial baseline for all future longitudinal studies in the region.

However, our study has primary limitations. First, our assay could not differentiate serotypes 6A and 6B. We analyzed this ambiguous serotype group separately to ensure transparent reporting. This molecular limitation means that the true dynamics of 6B, a key vaccine target, remain unknown in this cohort. Although this ambiguous group constitutes a small fraction of the total samples (a total of 20 samples were detected as dominant across all time points), the inability to resolve 6A (a non-PCV10) from 6B (a PCV10 target) means we cannot precisely quantify PCV10’s direct impact on 6B. This ambiguity limits the precise calculation of serotype replacement magnitude and may underestimate the true decline of PCV10 VTs. Therefore, the precise magnitude of replacement should be interpreted with caution. Future surveillance with methods that can distinguish these serotypes is crucial.

Second, the study was conducted entirely during the COVID-19 pandemic (2020–2022). Public health measures likely altered transmission dynamics, meaning our findings may not be fully representative of a non-pandemic period. Pandemic-related challenges also likely exacerbated participant attrition, increasing the risk of selection bias.

Third, we experienced loss to follow-up. While baseline characteristics did not differ significantly between retained and lost participants, bias from unmeasured factors cannot be entirely excluded. Finally, irregular evaluation intervals, due to logistic constraints, may have missed early acquisition events.

## 5. Conclusions

This study demonstrates a clear shift in pneumococcal ecology among Nepalese infants post-PCV10, with non-vaccine serotypes now dominating the nasopharynx. This reality necessitates a multi-pronged evolution in public health strategy. Methodologically, surveillance must adopt sensitive molecular techniques to accurately track the ongoing serotype replacement. Our findings provide a clear mandate to look beyond the vaccine vial towards environmental improvements, such as promoting better household ventilation. Strategically, these results underscore the urgent need to accelerate the broader introduction of higher-valence PCVs. A combined approach of advanced surveillance, environmental health, and next-generation vaccines will be essential to sustain long-term reductions in pneumococcal disease.

## Figures and Tables

**Table 1:** Serotypes covered by PCV10, PCV13, PCV15, and PCV20 vaccines.

Serotypes Included in Vaccines	4	6B	9V	14	18C	19F	23F	1	5	7F	3	6A	19A	22F	33F	8	10A	11A	12F	15B
PCV10	*	*	*	*	*	*	*	*	*	*										
PCV13	*	*	*	*	*	*	*	*	*	*	*	*	*							
PCV15	*	*	*	*	*	*	*	*	*	*	*	*	*	*	*					
PCV20	*	*	*	*	*	*	*	*	*	*	*	*	*	*	*	*	*	*	*	*

Background colors indicate vaccine serotype coverage: blue: PCV10 serotypes; red: additional PCV13 serotypes; light pink: additional PCV15 serotypes; purple: additional PCV20 serotypes. * denotes serotypes included in the respective pneumococcal conjugate vaccine.

**Table 2:** Characteristics of babies at enrolment.

Characteristics	*N* = 594 (%)	Characteristics	n = 594 (%)
Gender		Maternal education	
Female	275 (46.3)	None	47 (8)
Male	319 (53.7)	Secondary school	308 (52)
Gestational age	39 (38–39)	Higher education	239 (40)
<37 weeks	7 (1.2)	Source of income	
≥37 weeks	583 (98.8)	Business	277 (46.6)
Birth weight (g)	3000 (2800–3400)	Agriculture	176 (29.6)
<2500	54 (9.2)	Professional	136 (22.9)
>2500	535 (90.8)	Others ‡	5 (0.8)
Age (months)		Household size Median * (IQR)	4 (3–6)
Median * (IQR)	1.5 (1.4, 1.6)	<5 members	329 (55.5)
Ethnicity		5–10 members	249 (42.0)
Newar	359 (60.4)	>10 members	15 (2.5)
Tamang	116 (19.5)	Age of siblings in the household * (IQR)	5 (7–10)
Chhetri	46 (7.7)	<5 years old	74 (25.6)
Brahman	20 (3.4)	5–9 years old	148 (51.2)
Magar	13 (2.2)	10–17 years old	52 (18.0)
Rai	10 (1.7)	>17 years old	12 (4.2)
Others ‡	30 (5.1)	Smoker in the family	
Religion		No	342 (57.8)
Hindu	467 (78.6)	Yes	250 (42.2)
Buddhist	112 (18.9)	Kitchen location	
Christian	8 (1.3)	In living room	132 (22.2)
Muslim	3 (0.5)	Separate room	462 (77.8)
Others	4 (0.7)	Type of delivery	
Maternal age Median * (IQR)	29 (26–32)	Vaginal Delivery	286 (48.1)
<20 years old	20 (3.4)	Cesarian section	308 (51.9)
20–35 years old	522 (87.9)	Feeding methods	
>35 years old	52 (8.7)	Exclusive breast feeding	428 (72.1)
		Non-exclusive breast feeding	166 (27.9)

(IQR): Interquartile Range. Data are n (%) or median * (IQR). Subcategory totals do not add up to 594 where there was missing data. ‡ No further information was available on data recorded as ‘other’ on the data set.

**Table 3:** Pneumococcal Carriage Prevalence.

Time Point	Total Samples (*N*)	Overall Carriage, n (%) [95% CI]	Multiple Carriage, n (%) * [95% CI]
6 Weeks	594	179 (30.1%) [26.4–33.8]	69 (40.7%) [33.3–48.1]
9 Months	428	235 (54.9%) [50.2–59.6]	109 (47.4%) [40.9–53.9]
12 Months	353	153 (43.3%) [38.1–48.5]	47 (33.1%) [25.3–40.9]

Data are presented as n (%) with 95% confidence intervals (CI). * Percentage for multiple carriage was calculated among pneumococcal-positive samples with successful serotyping results (denominator: n = 172 at 6 weeks; n = 230 at 9 months; n = 142 at 12 months).

**Table 4:** Risk factors associated with pneumococcal carriage.

	n = 1375	Carriage Positive, n (%)	Carriage Negative, n (%)	OR (95% CI)	Adjusted OR (95% CI) *
Gender					
Female	748	299 (40.0)	449 (60.0)	1.1 (0.88–1.39)	1.1 (0.83–1.37)
Male	626	266 (42.5)	360 (57.5)	Ref	Ref
Age					
6 weeks	594	178 (29.9)	416 (70.0)	Ref	Ref
9 months	427	234 (54.8)	193 (45.2)	2.8 (2.19–3.64)	3.4 (2.6–4.55)
12 months	353	153 (43.3)	200 (56.7)	1.8 (1.39–2.37)	2.0 (1.47–2.69)
Kitchen location					
In living room	292	136 (46.6)	156 (53.4)	1.3 (0.99–1.74)	1.4 (1.00–1.87)
Separate room	1082	429 (39.7)	653 (60.3)	Ref	Ref
Seasons					
Pre-monsoon	287	129 (44.9)	158 (55.1)	1.7 (1.22–2.44)	2.0 (1.47–2.79)
Monsoon	330	106 (32.1)	224 (67.9)	Ref	Ref
Post-Monsoon	247	88 (35.6)	159 (64.4)	1.1 (0.79–1.64)	0.9 (0.57–1.13)
Winter	362	171 (47.2)	191 (52.8)	1.9 (1.35–2.60)	1.7 (1.47–2.69)

Data are n (%). Subcategory totals do not add up to 1375 where there was missing data. OR: Odds Ratio. * Adjusted for age, gender, kitchen location and seasons. CI: Confidence Interval. All odds ratios and confidence intervals were calculated from models weighted by Inverse Probability Weighting (IPW) to account for loss to follow-up.

**Table 5:** Risk factors associated with multiple serotype carriage.

	n = 567	Multiple Serotype Carriage, n (%)	Single Serotype Carriage, n (%)	OR (95% CI)	Adjusted OR (95% CI) *
Gender					
Female	285	112 (39.3)	173 (60.7)	1.2 (0.83–1.69)	1.2 (0.78–1.71)
Male	259	113 (43.6)	146 (56.4)	Ref	Ref
Age					
6 weeks	172	69 (40.1)	103 (59.9)	Ref	Ref
9 months	230	109 (47.4)	121 (52.6)	1.3 (0.87–1.95)	1.7 (1.06–2.68)
12 months	142	47 (33.1)	95 (66.9)	0.7 (0.47–1.18)	1.0 (0.61–1.70)
Kitchen location					
In living room	128	52 (40.6)	76 (59.4)	0.9 (0.58–1.39)	0.9 (0.56–1.43)
Separate room	416	173 (41.6)	243 (58.4)	Ref	Ref
Seasons					
Pre-monsoon	125	61 (48.8)	64 (51.2)	1.3 (0.74–2.15)	1.5 (0.89–2.79)
Monsoon	103	43 (41.7)	60 (58.3)	Ref	Ref
Post-Monsoon	84	33 (39.3)	51 (60.7)	0.9 (0.49–1.56)	0.8 (0.44–1.43)
Winter	164	62 (37.8)	102 (62.2)	0.8 (0.50–1.39)	1.0 (0.58–1.62)

Data are n (%). Subcategory totals do not add up to 567 where there was missing data. OR: Odds Ratio. * Adjusted for age, gender, kitchen location and seasons. CI: Confidence Interval. All odds ratios and confidence intervals were calculated from models weighted by Inverse Probability Weighting (IPW) to account for loss to follow-up.

## Data Availability

To protect participant confidentiality, the raw data from this study cannot be shared publicly. However, de-identified data may be made available upon reasonable request, pending review and approval by the appropriate ethics committee. Researchers interested in accessing the data should contact the corresponding author.

## Supplementary Materials

The following supporting information can be downloaded at: https://www.mdpi.com/article/10.3390/vaccines14010073/s1. References [39,40] are cited in the Supplementary Materials.

## Abbreviations

aOR	Adjusted Odds Ratio
CI	Confidence Interval
COVID-19	Coronavirus Disease 2019
Ct	Cycle threshold
GE/mL	Genome Equivalents per milliliter
GEE	Generalised estimating equation
IPW	Inverse Probability Weighting
IQR	Interquartile Range
LMICs	Low- and Middle-Income Countries
NVT	Non-Vaccine Type
ODK	Open Data Kit
PCV	Pneumococcal Conjugate Vaccine
PCV10	10-valent Pneumococcal Conjugate Vaccine
qPCR	Quantitative Polymerase Chain Reaction
SMH	Siddhi Memorial Hospital
STGG	Skim Milk, Tryptone, Glucose, and Glycerol
VT	Vaccine Type

## Appendix A

**Table A1:** Comparison of Baseline Characteristics Between Participants Retained and Lost to Follow-Up in a Longitudinal Study of Nepalese Infants (N = 594).

Variables	Retained (*n* = 277)	Lost to Follow-up (*n* = 317)	*p*-Value
Gender			
Female	127 (45.9%)	148 (46.7%)	0.84
Male	150 (54.2%)	169 (53.3%)	
Gestational age			
<37 weeks	3 (1%)	4 (1%)	0.84
≥37 weeks	272 (99%)	311 (99%)	
Birth weight (g)			
<2500	25 (9%)	29 (9%)	0.95
>2500	250 (91%)	285 (91%)	
Ethnicity			
Newar	172 (62%)	187 (59%)	0.95
Tamang	51 (18%)	65 (21%)	
Chhetri	19 (7%)	27 (9%)	
Brahman	10 (4%)	10 (3%)	
Magar	7 (3%)	6 (2%)	
Rai	5 (2%)	5 (2%)	
Others ‡	13 (5%)	17 (5%)	
Religion			
Hindu	221 (80%)	246 (78%)	0.59
Buddhist	48 (17%)	64 (20%)	
Christian	3 (1%)	5 (2%)	
Muslim	2 (1%)	1 (0%)	
Others ‡	3 (1%)	1 (0%)	
Maternal age			
<20 years old	8 (3%)	12 (4%)	0.83
20–35 years old	245 (88%)	277 (87%)	
>35 years old	24 (9%)	28 (9%)	
Maternal educational attainment			
Lower educational attainment	156 (56%)	199 (63%)	0.11
Higher educational attainment	121(44%)	118 (37%)	
Source of income			
Business	129 (47%)	148 (47%)	0.06
Agriculture	76 (27%)	100 (32%)	
Professional	72 (26%)	64 (20%)	
Others	0 (0%)	5 (1%)	
Household size			
<5 members	142 (51%)	187 (59%)	0.05
>5 members	135 (49%)	129 (41%)	
Age of siblings in the household			
<5 years old	32 (22%)	42 (30%)	0.1
>5 years old	115 (78%)	97 (70%)	
Presence of smoker in the family			
No	160 (58%)	182 (58%)	0.99
Yes	117 (42%)	133 (42%)	
Kitchen location			
In living room	58 (21%)	74 (23%)	0.48
Separate room	219 (79%)	243 (77%)	
Type of delivery			
Vaginal Delivery	128 (46%)	158 (50%)	0.38
Lower segment Cesarian section	149 (54%)	159 (50%)	
Feeding methods			
Exclusive breast feeding	192 (69%)	236 (74%)	0.16
Non-exclusive breast feeding	85 (31%)	81 (26%)	

IQR: Interquartile Range. Data are n (%) or median (IQR). Subcategory totals do not add up to 594 where there was missing data. ‡ No further information was available on data recorded as ‘other’ on the data set.

**Table A2:** Pneumococcal serotypes distribution at 6 weeks, 9 months, and 12 months of age.

Vaccines and Included Serotypes	6 Weeks (n = 179)	9 Months (n = 235)	12 Months (n = 153)	*p*-Value
PCV10				
1		1 (0.4)		
4	1 (0.6)	2 (0.9)		0.53
5		1 (0.4)		
7F/7A		1 (0.4)		
9V/9A	4 (2.2)	1 (0.4)		0.05
14	2 (1.1)	1 (0.4)		0.36
18F/18A/18B/18C		3 (1.3)		
19F	78 (43.6)	76 (32.3)	16 (10.5)	<0.001
23F	6 (3.4)	4 (1.7)	2 (1.3)	0.37
6A/6B Serotype group				
6A/6B	6 (3.4)	13 (5.5)	6 (3.9)	0.53
PCV13				
3	8 (4.5)	12 (5.1)	11 (7.2)	0.53
19A	16 (8.9)	29 (12.3)	12 (7.8)	0.29
PCV15				
22F/22A	20 (11.2)	6 (2.6)	5 (3.3)	<0.001
33F/33A/37		2 (0.9)		
PCV20				
10A	2 (1.1)	10 (4.3)	6 (3.9)	0.16
11A/11D/11E/11F	3 (1.7)	16 (6.8)	9 (5.9)	0.04
12F/12A/12B/44/46	7 (3.9)	1 (0.4)	1 (0.7)	0.01
15B/15C	14 (7.8)	19 (8.1)	19 (12.4)	0.26
Other serotypes				
2	1 (0.6)			
6C/6D	8 (4.5)	20 (8.5)	19 (12.4)	0.03
7C	1 (0.6)	3 (1.3)	1 (0.7)	0.69
9N/9L	9 (5.0)	1 (0.4)	2 (1.3)	<0.001
10F/10C	5 (2.8)			
13	3 (1.7)	6 (2.6)	3 (1.9)	0.82
15F/15A	7 (3.9)	9 (3.8)	11 (7.2)	0.26
16F	4 (2.2)	2 (0.9)	3 (1.9)	0.49
17F	1 (0.6)	2 (0.9)	2 (1.3)	0.77
20	1 (0.6)	2 (0.9)	1 (0.7)	0.94
21	1 (0.6)	1 (0.4)	1 (0.7)	0.94
23A	4 (2.2)	17 (7.2)	4 (2.6)	0.02
23B	8 (4.5)	10 (4.3)	10 (6.5)	0.56
24F/24A/24B/24C	5 (2.8)	14 (6.0)	3 (2.0)	0.09
31		6 (2.6)		
33F/33A/37		2 (0.9)		
34	10 (5.6)	15 (6.4)	9 (5.9)	0.94
35A/35C/42	2 (1.1)	6 (2.6)	4 (2.6)	0.53
35B	19 (10.6)	42 (17.9)	27 (17.7)	0.9
35F/47	1 (0.6)	1 (0.4)		0.67
37	1 (0.6)	1 (0.4)		0.53
38F/25F/25A	2 (1.1)	1 (0.4)		0.36
39		2 (0.9)		
Non-typeable	4 (2.2)	9 (3.8)	7 (4.6)	0.49
