# Supplementary material for: Non-Vaccine Serotype Replacement and Subdominant Persistence of Vaccine Types in Nepalese Infants Following PCV10 Introduction
Source: Vaccines (Basel). 2026 Jan 8;14(1):73. doi: 10.3390/vaccines14010073 (PMC12846638; doi:10.3390/vaccines14010073)
Supplement: Supplementary file 1 [file vaccines-14-00073-s001.zip › vaccines-4066861-supplementary.pdf]

Supplementary material Methods

Table of Contents

Methods ... 2

Laboratory analysis ... 2

Detailed Description of Logistic Regression Model Building ... 3

Methods

Laboratory analysis

Sample Collection and Processing

Nasopharyngeal swabs were collected from participants according to the World Health Organization's standardized guidelines for nasopharyngeal sampling [14]. Immediately following collection, each swab was placed into a sterile tube containing 1 mL of Skim Milk, Tryptone, Glucose, and Glycerol (STGG) transport medium [14]. This medium is specifically formulated to maintain the viability and integrity of pneumococcal bacteria during storage and transport [14]. The tubes were then placed in a cool box maintained at a temperature of 4°C to minimize bacterial degradation. Within 8 hours of collection, all samples were transported to the laboratory at Siddhi Memorial Hospital (SMH) and stored at -80°C until further processing.

DNA Extraction

DNA extraction was performed at SMH using the QIAamp DNA Mini Kit (Qiagen, Hilden, Germany) following the manufacturer's instructions. This kit utilizes a spin-column based method to isolate high-quality DNA from the nasopharyngeal samples. Briefly, the samples were thawed and subjected to enzymatic lysis to release the bacterial DNA. The lysate was then passed through a silica membrane column, where the DNA binds selectively. Impurities were removed through a series of wash steps, and the purified DNA was finally eluted in a designated buffer. The extracted DNA samples were then transported on dry ice to the Institute of Tropical Medicine, Nagasaki University (NU) and stored at -80°C until further analysis.

Pneumococcal Detection

At NU, quantitative real-time PCR (qPCR) was employed to detect the presence of pneumococcal DNA. This technique targets the lytA gene, a highly conserved gene specific to Streptococcus pneumonia [11]. The qPCR assay was performed using validated primers and probes, and a positive result was defined as a cycle threshold (Ct) value below 30 [11]. This threshold was chosen based on previous studies demonstrating its high sensitivity and specificity for detecting pneumococcal DNA in clinical samples [11].

Molecular Serotyping

Molecular serotyping was performed using the X9 nanofluidic real-time PCR system (Fluidigm). This advanced technology allows for the simultaneous detection of up to 70 different pneumococcal serotypes in a single reaction. The assay follows a previously described method [11], which involves two main steps:

Specific Target Amplification (STA):

In this initial amplification step, pooled primers targeting serotype-specific genes are used to amplify the target DNA sequences. The reaction is performed for 14 cycles using TaqMan PreAmp Master Mix (Applied Biosystems), which contains optimized reagents for preamplification. Following STA, the amplified product is diluted to prepare it for the next step [11].

Nanofluidic PCR:

The diluted STA product is loaded onto a Fluidigm 48.48 Dynamic Array IFC chip. This chip contains microfluidic channels that allow for precise partitioning of the sample and reagents, enabling high-throughput real-time PCR analysis. Serotype-specific TaqMan probes are used to detect the amplified DNA sequences [11]. Fluorescence signals generated during the PCR reaction are monitored in real-time, and melting curve analysis is performed to confirm the specificity of the amplified products [11]. The presence or absence of each serotype is determined based on the fluorescence signals and melting curve profiles.

Detailed Description of Logistic Regression Model Building

Candidate Variable Selection:

To identify independent risk factors for pneumococcal carriage, we constructed a multivariable logistic regression model using Generalized Estimating Equations (GEE) and a purposeful selection strategy [40]. Based on comprehensive review of existing literature, age and gender were identified as a priori confounders. These two variables were forced into the multivariable model regardless of their p-value to ensure a valid and properly adjusted final model.

Other candidate variables were identified from the literature, including maternal characteristics (age, education level), household characteristics (kitchen location, age of siblings, household size, ethnicity), and seasonal factors. For this analysis, seasons were categorized based on four different patterns: pre-monsoon (March–May), monsoon (June–September), post-monsoon (October–November), and winter (December–February). These variables were first screened individually using univariable logistic regression. To avoid prematurely excluding important confounders, we used a lenient screening threshold ($p < 0.25$) [40]to identify all potential candidates for the multivariable model.

An initial multivariable model was then built containing the a priori confounders (age, gender) plus all candidate variables that passed the screening. We assessed this model for multicollinearity using the Variance Inflation Factor (VIF). For example, age of siblings was highly correlated with the infant's age, which introduced model instability (wide confidence intervals) without improving the model's fit; it was therefore excluded from the final model.

We then refined the model by iteratively removing non-significant variables ($p > 0.15$) [40], provided they were not the a priori confounders and their removal did not significantly change the odds ratios of the remaining key predictors.

To select the most appropriate working correlation structure for the GEE model, we compared different structures (independent, exchangeable, unstructured) using the Quasi-likelihood Information Criterion (QIC) [41]. The exchangeable structure yielded the lowest QIC value (QIC = 298.339), indicating the best relative fit compared to the unstructured structure (QIC = 305.123), and was therefore used for the final model.

Similarly, to identify risk factors for multiple serotype carriage (defined as carrying >1 serotype), we built a separate multivariable logistic GEE model (family(binomial), link(logit)), forcing in age and gender as a priori confounders and screening other candidate variables ($p < 0.25$) [40].

We compared different working correlation structures for the multiple carriage model using QIC [41]. The exchangeable structure yielded the lowest QIC value indicating the best relative fit, and was therefore used for the final multiple carriage model
